# Prognostic and Predictive Significance of Cancer Stem Cell Markers (ZIP-4 and ALDH1A1) in Ovarian Serous Carcinoma Patients: An Immunohistochemical Study

**DOI:** 10.31557/APJCP.2026.27.1.255

**Published:** 2026-01-22

**Authors:** Amany Selim Attia, Mohamed Abdalsalam, Ahmed S. E. M. Iraki, Samia Hussein, Ahmad Barakat Waley, Amr Khalil, Zahraa I. Aboafya, Reham Sameh

**Affiliations:** 1 *Department of Pathology, Faculty of Medicine, Zagazig University, Sharkia, Egypt.*; 2 *Department of Obstetrics& Gynaecology, Al-Ahrar Teaching Hospital, Sharkia, Egypt.*; 3 *Department of Obstetrics& Gynaecology, Faculty of Medicine, Zagazig University, Sharkia, Egypt.*; 4 *Department of Medical Biochemistry and Molecular Biology, Faculty of Medicine, Zagazig University, Sharkia, Egypt.*; 5 *Department of Medical Oncology, Faculty of Medicine, Zagazig University, Sharkia, Egypt.*; 6 *Department of Surgical Oncology, Al-Ahrar Teaching Hospital, General Organization for Teaching Hospitals and Institutes (GOTHI), Egypt.*; 7 *Department of Clinical and Chemical Pathology, Institute of Medical Research and Clinical Studies, National Research Centre, Cairo, Egypt.*

**Keywords:** ZIP-4, ALDH1A1, Ovarian cancer

## Abstract

**Background:**

Worldwide, ovarian cancer is the eighth most common cancer among females and the fifth leading cause of cancer-related deaths in women. In Egypt, it accounts for 4.5% of all cancer cases and ranks the fourth most common cancer among women. Cancer stem cells (CSCs) play a crucial role in tumor growth and chemoresistance. Our study examined the expression of cancer stem cell markers (ZIP-4 and aldehyde dehydrogenase-1 member A1 (ALDH1A1)) in ovarian serous carcinoma tissues using immunohistochemistry. We also analyzed the relationship between their expression levels and clinicopathological features, patient survival, and response to platinum-based chemotherapy.

**Subjects & Method:**

This study included 55 patients with ovarian serous carcinoma. Immunohistochemical staining for ZIP-4 and ALDH1A1 was performed.

**Results:**

Statistically significant relationships were detected between high ZIP-4 and ALDH1A1 expressions and patient age, tumor size, presence of malignant ascites, lymphovascular invasion, elevated cancer antigen-125 (CA-125) levels, disease stage, and lymph node involvement (P < 0.001 for each). Additionally, the log-rank test showed that high ZIP-4 and ALDH1A1 expressions were associated with shorter disease-free survival (DFS) (P = 0.002 and <0.001, respectively) and overall survival (OS) (P < 0.001 for each).

**Conclusion:**

Ovarian cancer stem cell markers (ZIP-4 and ALDH1A1) can be considered potential prognostic markers in ovarian cancer patients. Moreover, ZIP-4 and ALDH1A1 expressions are related to resistance to platinum-based chemotherapy, which leads to ovarian serous carcinoma progression. Clinical implications suggest that future therapeutic regimens targeting ZIP-4 and ALDH1A1 may help overcome platinum-based chemotherapy resistance and improve patients outcomes.

## Introduction

Ovarian cancer is a lethal malignancy in females. It is the eighth most common cancer among females and the fifth leading cause of cancer mortality among them [1]. In Egypt, ovarian cancer represents 4.5% of all cancer cases, and it is the fourth most common cancer in women [2]. 

Epithelial ovarian carcinoma represents 95% of ovarian malignancies [3]. Serous ovarian carcinoma, one of the histological subtypes of epithelial ovarian carcinoma, has a high mortality rate and poor outcome [4]. Poor outcome is explained by late diagnosis at advanced stages, high tumor recurrence, and chemotherapy resistance. Thus, it is crucial to identify potential predictive biomarkers for tumor progression, recurrence, and chemotherapy resistance, which can lead to improvements in patient diagnosis and outcomes [5].

Tumor development and treatment resistance in many cancers can be explained by cancer stem cells (CSCs). These cells are characterized by self-renewal, multipotency, and the ability to differentiate into several cells that are responsible for carcinogenesis [6, 7]. Ovarian CSCs can be responsible for cancer growth, progression, metastasis, recurrence, and chemoresistance. They can be a potential targeted ovarian cancer therapy [8].

ZIP-4 is a zinc transporter. It is a cancer-related protein in many tumors [9-12]. Additionally, it is responsible for activities related to cancer stem cells [13]. Aldehyde dehydrogenase-1 member A1 (ALDH1A1) has been found in many neoplastic and non-neoplastic tissues. ALDH1A-positive tumor cells have cancer stem properties. Additionally, its upregulation in many cancers was linked to tumor invasiveness, proliferation, neo-angiogenesis, chemoresistance, and poor survival. Furthermore, ALDH1A1 inhibition led to increased tumor chemosensitivity. However, ALDH1A1 expression in ovarian cancers exhibited conflicting results, where serous carcinoma was associated with poor prognosis, while other types showed opposite results [14].

In our study, ZIP-4 and ALDH1A1 were selected over other cancer stem cell markers because they are well-established ovarian cancer stem cell markers. ZIP-4 promotes tumor progression and enhances stemness properties [13], while ALDH1A1 identifies ovarian CSCs and directly correlates to platinum resistance [15]. Additionally, ZIP-4 is an upstream regulator of ALDH1A1. ZIP-4 upregulation increased ALDH1A1 expression, suggesting a functional link between the two pathways that reinforces their combined role in ovarian cancer aggressiveness [13]. 

In our study, we hypothesized that the expression of cancer stem cell markers (ZIP-4 and ALDH1A1) is associated with aggressive clinicopathological features, poorer survival, and chemotherapy resistance in ovarian serous carcinoma. 

## Materials and Methods

This prospective cohort study included 55 ovarian serous carcinoma patients who were admitted to Zagazig University Hospital, Faculty of Medicine, Zagazig University and Al-Ahrar Teaching Hospital, Sharkia, Egypt, from March 2022 to September 2022. Follow-up was performed for 3 years. All patients were subjected to a comprehensive history taking and a thorough physical examination. The tumor marker cancer antigen-125 (CA-125) measurement was performed, along with a contrast-enhanced CT scan, MRI, or PET scan.

The patients underwent optimal surgical staging/debulking at the Obstetrics and Gynecology Department, Faculty of Medicine, Zagazig University and Al-Ahrar Teaching Hospital, Sharkia, Egypt. They received their adjuvant platinum-based chemotherapy with or without neoadjuvant therapy at the Medical Oncology Department, Faculty of Medicine, Zagazig University, Egypt. Six cycles of platinum-based intravenous (IV) chemotherapy were recommended for patients with stages I (high grade) and II–IV. Paclitaxel 175 mg/m^2^ IV was given first, followed by carboplatin AUC 5–6 given IV over 30–60 minutes. This regimen was repeated every 3 weeks. Patients were switched to a second-line treatment if they had signs of progressive disease or inadequate responses. Every 2 to 3 chemotherapy cycles, CA-125 levels and contrast-enhanced CT/MRI or PET CT scans were used to evaluate responses to treatment. 

Detailed information was obtained from the participants, including age, postoperative histopathological diagnosis, histological type, stage according to the International Federation of Gynecology and Obstetrics (FIGO) (8th edition), tumor size, lymph node metastasis, distant metastasis, ascites cytology result, chemotherapy regimen, type of surgical operation, serum CA-125 level, recurrence status, disease-free survival (DFS), progression-free survival (PFS), and overall survival (OS). DFS was calculated from the start of treatment to the time of relapse or the last follow-up visit as relapse-free. PFS was calculated as the time from the beginning of treatment to disease progression or the last follow-up visit, as progression-free. OS was calculated as the time from diagnosis to death or the last follow-up contact (censored). Distant metastasis was judged by diagnostic imaging. The study got approval from the Institutional Review Board of the Faculty of Medicine, Zagazig University, Egypt (ZU-IRB# 432/2-June-2024).

The included cases were those with definite histopathological confirmation of ovarian serous carcinoma and fulfilled clinical information. The excluded cases were of inadequate tissue material, benign tumors, borderline tumors, undifferentiated carcinomas, tumors of non-epithelial origin, metastatic tumors, incomplete clinical data other non serous epithelial tumors, and cases with massive necrosis and fibrosis. Histopathological evaluation was performed by two blinded pathologists to minimize bias.

CA-125 measurement was performed by obtaining venous blood samples from patients. After serum preparation, CA-125 was measured using the Elecsys CA 125 II assay, which uses electrochemiluminescence (ECLIA) technology. This assay is performed on an automated Cobas e immunoassay analyzer. CA-125 measurement was performed initially at admission and frequently in follow-up. 

Histopathological evaluation was performed according to the criteria of the World Health Organization (WHO) classification of tumors of the female reproductive organs. Four-micron-thickness sections were used. Staining with hematoxylin &eosin (H&E) was performed to confirm the diagnosis and tumor grade. Staining with ZIP-4 antibody (Proteinntech, dilution 1:500) and ALDH1A1 monoclonal antibody (EP1933Y, diluted at 1:200-400) was performed to detect their immunohistochemical (IHC) expressions. Using the Dako Autostainer following the instructions of the manufacturer.

Immunohistochemical staining was evaluated by a semiquantitative scoring method. ZIP-4 positivity was recognized by brownish cytoplasmic coloration. ZIP-4 was scored into no staining (0), light positive staining (1), medium positive staining (2), and strong positive staining (3). The area of positive staining was scored into <5% (0), 5–25% (1), 26–50% (2), 51–75% (3), and >75% (4). Overall scoring was obtained by multiplying the intensity and expression scores for each sample. ZIP-4 expression was classified into high or low according to the median [16].

ALDH1A1 positivity was detected by brownish cytoplasmic coloration. Additionally, it was semi-quantitatively scored according to positive tumor cells as 0 (<5%), 1 (5-20%), 2 (21 to 50%), and 3 (>51%) with subsequent classification into 2 groups: low expression (scores 0 and 1) or high expression (scores 2 and 3) [17].

*Statistical analysis*

SPSS 22.0 for Windows (IBM Corp.) was used for statistical analysis. Continuous variables were expressed as mean±SD and median (range), and the categorical variables were presented as a number (percentage). Percentages of categorical variables were compared using the X^2^ test or Fisher’s exact test as appropriate. Stratification of DFS, PFS, and OS rates was estimated using a Kaplan-Meier plot and compared using the log-rank test. All tests were two-sided. A P-value < 0.05 was considered significant.

## Results

### Clinicopathological Features and IHC expression

**Figure 1:**
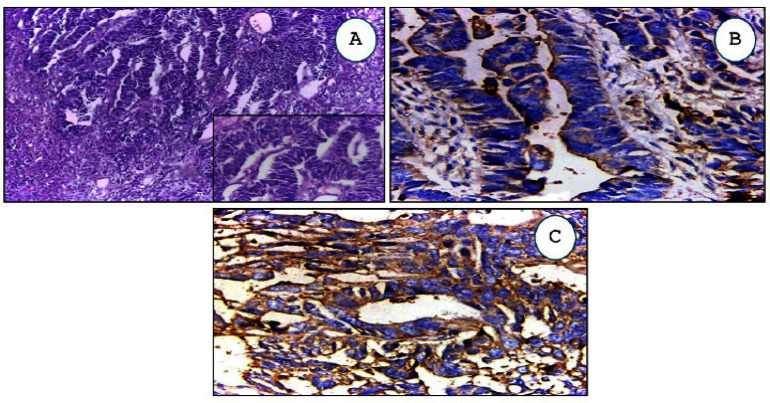
A: low-grade ovarian serous carcinoma with mild to moderate atypia with low mitotic activity. It occasionally forms papillary structures (x100 HPF) with attached high-power insert (x400 HPF); B: low-grade ovarian serous carcinoma with low ZIP4 cytoplasmic expression (x400 HPF); C: low-grade ovarian serous carcinoma with low ALDH1A cytoplasmic expression (x400 HPF).

**Figure 2:**
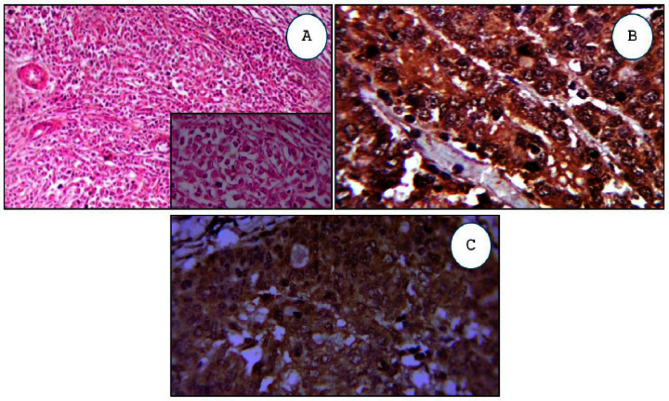
A: High-grade ovarian carcinoma with marked nuclear atypia and solid pattern (x100 HPF) with attached high-power insert (x400 HPF); B: High-grade ovarian serous carcinoma with high ZIP4 cytoplasmic expression (x400 HPF); C: High-grade ovarian serous carcinoma with high ALDH1A cytoplasmic expression (x400 HPF).

At diagnosis, the patients’ ages ranged from 41 to 75 years, with a mean of 58.7±9.9 years. About 67.3% of the studied patients were ≥ 50 years old, 12.7% had positive family history, 61.8% had tumor size > 5 cm, 61.8% had high-grade tumor, and 69.1% showed positive lymph node metastasis. Lymphovascular invasion occurred in 45.5% of the enrolled cases. Regarding staging, 14.5%, 16.4%, 47.3%, and 21.8% had stage I, II, III, and IV, respectively. Distant metastasis was observed in 21.8% of cases (Table 1). Low ZIP-4 expression was detected in 47.3% of patients (Figure 1B), while high ZIP-4 expression was observed in 52.7% of cases (Figure 2B). 36.4% of patients showed low ALDH1A1 expression (Figure 1C), while high ALDH1A1 expression was observed in 63.6% of cases (Figure 2C). 

### Association between ZIP-4 and ALDH1A1 expression and clinicopathological parameters

Statistically significant relationships were detected between high ZIP-4 and ALDH1A1 expressions and each of higher age of the studied patients, increased tumor size, presence of malignant ascites, lymphovascular invasion, high CA-125 level, advanced stage, and lymph node metastasis (P < 0.001 for each), higher tumor grades (P = 0.001 and < 0.001, respectively), positive family history (P = 0.02 and 0.004, respectively), and the presence of distant metastasis (P = 0.002 and 0.02, respectively) (Table 2). 

### Association between ZIP-4 and ALDH1A1 expression and response to chemotherapy

Compared with patients with low expressions, all patients with high ZIP-4 and high ALDH1A1 expression received chemotherapy (P=0.01 and 0.002, respectively). The majority of them showed a progressive disease course (P<0.001 for each), higher relapse (P=0.01 and <0.001, respectively), and higher mortality (P<0.001 for each). 

The progressive disease course was significantly higher among patients with high ZIP-4 expression compared to those with low expression (82.6% versus 11.1%), while stable and progressive disease courses were found to be significantly higher among patients with high ALDH1A1 expression compared to those with low expression (25%, 67.9% versus 0% and 25%, respectively). Additionally, relapse and mortality rates were significantly higher among patients with high ZIP-4 expression compared to those with low expression (83.5%, 93.1% versus 26.3% and 19.2%, respectively), and among patients with high ALDH1A1 expression compared to those with low expression (100%, 82.9% versus 16.7% and 15%, respectively). Platinum-resistant relapse was significantly higher among those with high ALDH1A1 expression (P<0.01) (Table 3).

### Association between ZIP-4 and ALDH1A1 expression and patients’ survival

**Figure 3:**
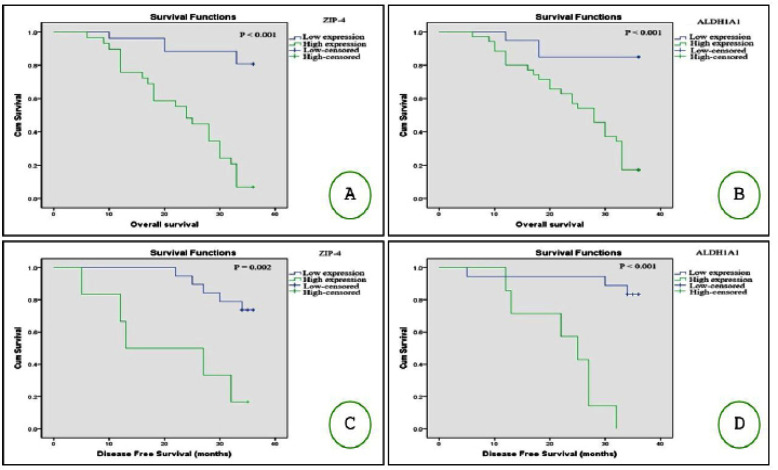
Kaplan-Meier Survival Analysis Curve. A: showing overall survival in relation to ZIP-4 marker; B: showing overall survival in relation to ALDH1A1 marker; C: showing disease-free survival in relation to ZIP-4 marker; D: showing disease-free survival in relation to ALDH1A1 marker

High ZIP-4 and ALDH1A1 expressions were significantly correlated with lower disease-free survival (DFS) (P=0.001 and 0.004, respectively) and lower overall survival (OS) (P<0.001 and 0.002, respectively). However, progression-free survival (PFS) didn’t show any significant differences regarding ZIP-4 or ALDH1A1 expression (P=0.257 and 0.252, respectively) (Table 4). Log-Rank test showed that high *ZIP-4* and *ALDH1A1* expressions were associated with shorter DFS (P=0.002 and P <0.001, respectively) and shorter OS (P <0.001, for both). (P<0.001, for each) (Figure 3, Table 5). 

## Discussion

Ovarian cancer stem cells exhibit resistance to chemotherapy. Additionally, they possess the ability to self-renewal, plasticity, and tumor regeneration. The tumor microenvironment maintains ovarian cancer stem cells by supplying nutrients and oxygen gradients, extracellular matrix interactions, and immune cell modulation. Additionally, cancer-associated fibroblasts produce growth factors and cytokines that create a pro-tumorigenic niche, promoting CSC maintenance, invasion, and chemoresistance. Moreover, several signaling pathways support CSC, including WNT, NOTCH, PI3K/AKT/mTOR, TGF-β, JAK/STAT, Hedgehog, NF-κB, and Hippo [18].

In the present study, we assessed the expression of CSC markers (ZIP-4 and ALDH1A1) in serous ovarian carcinoma. High ZIP-4 expression was observed in 52.7% of studied cases. Statistically significant relationships were detected between high ZIP-4 expression and increased tumor size, presence of distant metastasis, presence of malignant ascites, lymphovascular invasion, higher CA-125 levels, advanced stage, higher tumor grades, and lymph node metastasis. Additionally, statistically significant relationships were detected between high ZIP-4 expression and chemotherapy resistance, high tumor relapse and mortality. Moreover, high ZIP-4 expression was associated with poor OS and DFS.

Similar results were found by Fan et al. [19, 20], where ZIP-4 was overexpressed in human epithelial ovarian cancer tissues (by immunoblotting, quantitative polymerase chain reaction, and immunohistochemical staining) compared to normal and benign tissues. Additionally, ZIP-4 overexpression increased tumorigenesis and chemoresistance in high-grade serous ovarian carcinoma. Furthermore, ZIP-4 knockdown significantly reduced cancer cell proliferation and drug resistance. Additionally, Fan et al. [13] reported that 75% of high-grade ovarian serous carcinoma samples showed ZIP-4 overexpression. Moreover, ZIP-4-positive cells exhibited self-renewal and differentiation potential and formed tumors and ascites in vivo. Furthermore, high ZIP-4 expression contributed to chemoresistance in vitro. 

The tumorigenic role of ZIP-4 can be explained by its interaction with the NOTCH3 pathway. ZIP-4 acts as an upstream regulator of NOTCH3, responsible for CSC-like activities and tumorigenesis in high-grade ovarian serous carcinoma. Thus, the ZIP-4-NOTCH3 pathway represents a possible therapeutic target in high-grade ovarian serous carcinoma [13].

Concerning ALDH1A1, our results showed that high ALDH1A1 expression was observed in 63.6% of cases. Statistically significant associations were found between high ALDH1A1 expression and higher CA-125 levels, high tumor grade, increased tumor size, presence of malignant ascites, lymphovascular invasion, lymph node metastasis, advanced tumor stages, and presence of distant metastasis. Additionally, statistically significant relationships were detected between high ALDH1A1 expression and chemotherapy resistance, high tumor relapse and mortality. Moreover, statistically significant relationships were found between high ALDH1A1 expression and lower DFS and OS.

Khalifa et al. [17] found similar results. They showed a positive relationship between ALDH1A1 overexpression and higher tumor grades. Additionally, Zhao et al. [21] found that ALDH1A1 was elevated in patients with poor clinicopathological criteria, and was associated with FIGO stage, lymph node involvement, and distant metastasis. They also found that high ALDH1A1 expression was significantly associated with poor OS . Another meta-analysis by Tao et al. [22] showed that high expression of ALDH1A1 was correlated with poor OS and DFS. Moreover, Ayub et al. [23] found that overexpression of ALDH1A1 in patients with advanced epithelial ovarian cancer after treatment was associated with poor response to chemotherapy . Additionally, Roy et al. [24] found that ALDH1A1 isoform expression in patients with high-grade ovarian serous carcinoma was associated with poor response to platinum-based therapy . Furthermore, Izycka et al. [14] found that positive ALDH1A1 expression can be considered an independent prognostic factor of shorter OS and PFS in patients with epithelial ovarian cancer . 

The tumorigenic role of ALDH1A1 in ovarian cancer can be explained by enhancing CSC properties. Additionally, they promote metastasis by altering metabolic pathways, enhancing angiogenesis, and inducing immune evasion. Moreover, they cause treatment resistance by detoxifying chemotherapeutic drugs and activating the Wnt/ β-catenin survival pathway. ALDH1A1-expressing ovarian cancer cells can maintain platinum-resistance by dysregulating the cell-cycle checkpoint and DNA repair network [15]. Furthermore, ALDH1A1 converts retinol to retinoic acid, which activates nuclear receptors to regulate gene expression, stemness, cell signaling, and DNA repair [25-29].

Frequent recurrence of ovarian cancer is an important therapeutic problem, even with an initial promising response. Additionally, resistance to chemotherapy can result in treatment failure or death. The mechanism of development of chemoresistance is possibly mediated by CSCs, which causes recurrence after chemotherapy. Chemoresistance of ovarian CSCs can also be caused by increased drug effects, CSCs quiescence, accelerated DNA repair, and autophagy [30]. The role of CSCs in metastasis in ovarian cancer is related to survival in non-adherent conditions and later adherence in non-primary sites and the creation of secondary tumors. Furthermore, plasticity of CSCs helps them to undergo epithelial-mesenchymal transition with subsequent metastasis [31]. 

Our results support the possibility of using the expression of cancer stem cell markers (ZIP-4 and ALDH1A1) in predicting ovarian cancer patients with platinum-resistance and poor prognosis. Targeting these markers may be a promising treatment strategy. Previous studies suggested targeting ZIP-4 via RNA interference and HDAC inhibitors [20]. Additionally, targeting ALDH1A1 via RNA interference, small-molecule inhibitors, or pan-ALDH1A family inhibitors was investigated, as well [26, 32].

In conclusion ovarian CSC markers (ZIP-4 and ALDH1A1) may be related to resistance to platinum-chemotherapy, which leads to ovarian serous carcinoma progression. So, therapeutic targets against both ZIP-4 and ALDH1A1 may have potential roles in overcoming platinum-resistance and improving outcomes.

### Limitations of the study

The relatively small sample size is one of the limitations of this research, making its results difficult to apply to the general population. Future multi-center cohort studies with increasing sample size are recommended. This allows multivariate analysis to adjust for confounders. Furthermore, exploring targeted therapies is highly recommended.

**Table 1:** Clinicopathological Features, immunohistochemical Eexpression, and Outcome of the Studied Patients.

	Studied group (n=55)			
Variable	No.		%	
Age				
Mean ±SD	58.7 ±9.9			
Range	41–75			
Age group				
<50 years	18		32.70%	
≥50 years	37		67.30%	
Family history				
Negative	48		87.30%	
Positive	7		12.70%	
Baseline CA-125				
Normal	16		29.10%	
High	39		70.90%	
Surgery				
Surgical staging	6		10.90%	
Optimal debulking	17		30.9%	
Sub-optimal debulking	32		58.2%	
Tumor laterality				
Unilateral	26		47.30%	
Bilateral	29		52.70%	
Tumor grade				
Low grade	21		38.20%	
High grade	34		61.80%	
Malignant ascites				
Absent	18		32.70%	
Present	37		67.30%	
Implants				
Absent	20		36.40%	
Present	35		63.60%	
Maximum tumor size				
≤5 cm	21		38.20%	
>5 cm	34		61.80%	
LVI				
Absent	30		54.50%	
Present	25		45.50%	
LN metastasis				
Negative	17		30.90%	
Positive	38		69.10%	
Distant metastasis				
Absent	43		78.20%	
Present	12		21.80%	
Residual disease				
Absent	23		41.80%	
Present	32		58.20%	
FIGO stage				
Stage IA	1		1.80%	
Stage IB	5		9.10%	
Stage IC	2		3.60%	
Stage IIA	4		7.30%	
		Studied group (n=55)			
Variable		No.		%	
FIGO stage					
Stage IIB		5		9.10%	
Stage IIIA		11		20%	
Stage IIIB		10		18.20%	
Stage IIIC		5		9.10%	
Stage IVA		9		16.40%	
Stage IVB		3		5.50%	
ZIP-4					
Low		26		47.30%	
High		29		52.70%	
ALDH1A1					
Low		20		36.40%	
High		35		63.60%	
ZIP-4/ALDH1A1					
Low/Low		16		29.10%	
High/Low		4		7.30%	
Low/High		10		18.20%	
High/High		25		45.50%	
Chemotherapy					
No		5		9.10%	
Yes		50		90.90%	
Response to treatment		(n=32)			
Complete response		3		9.40%	
Partial response		2		6.30%	
Stable Disease		7		21.80%	
Progressive Disease		20		62.50%	
Follow-up duration (months)					
Mean ± SD		27.95±9.56			
Median (Range)		33 (6-36)			
Relapse					
Absent		15		27.30%	
Present		10		18.20%	
Type of relapse					
Platinum sensitive		4		7.30%	
Platinum resistance		6		10.90%	
Progression					
Absent		19		34.50%	
Present		36		65.50%	
Mortality					
Alive		23		41.80%	
Died		32		58.20%	

Categorical variables were expressed as number (percentage). Continuous variables were expressed as mean ± SD & median (range). FIGO, International Federation of Gynecology and Obstetrics; LVI, lymph-vascular invasion.

**Table 2.** Relationship between ZIP-4 and ALDH1A1 Expression and Clinicopathological Features of the Studied Patients

	All	ZIP4 expression				ALDH1A1 expression			
Variable	(n=55)	Low (n=26)	High (n=29)	Test	p-value	Low (n=20)	High (n=35)	Test	p-value
Age group									
<50 years	18 (32.7%)	16 (61.5%)	2 (6.9%)	18.59	<0.001**	15 (75%)	3 (8.6%)	25.5	<0.001**
≥50 years	37 (67.3%)	10 (38.5%)	27(93.1%)			5 (25%)	32(91.4%)		
Family history									
Negative	48 (87.3%)	20 (76.9%)	28(96.6%)	4.755	0.02*	14 (70%)	34(97.1%)	8.442	0.004*
Positive	7 (12.7%)	6 (23.1%)	1 (3.4%)			6 (30%)	1 (2.9%)		
CA-125									
Normal	16 (29.1%)	14 (53.8%)	2 (6.9%)	14.64	<0.001**	15 (75%)	1 (2.9%)	31.11	<0.001**
High	39 (70.9%)	12 (46.2%)	27(93.1%)			5 (25%)	34(97.1%)		
Tumor grade									
Low grade	21 (38.2%)	18 (69.2%)	3 (10.3%)	20.14	0.001**	17 (85%)	4(11.4%)	29.18	<0.001**
High grade	34 (61.8%)	8 (30.8%)	26(89.7%)			3 (15%)	31(88.6%)		
Tumor laterality									
Unilateral	26 (47.3%)	23 (88.5%)	3 (10.3%)	33.56	<0.001**	18 (90%)	8 (22.9%)	23.01	<0.001**
Bilateral	29 (52.7%)	3 (11.5%)	26(89.7%)			2 (10%)	27 (77.1%)		
Malignant ascites									
Absent	18 (32.7%)	16 (61.5%)	2 (6.9%)	18.59	<0.001**	15 (75%)	3 (8.6%)	25.5	<0.001**
Present	37 (67.3%)	10 (38.5%)	27(93.1%)			5 (25%)	32 (91.4%)		
Maximum tumor size									
≤5 cm	21 (38.2%)	18 (69.2%)	3 (10.3%)	20.14	<0.001**	17 (85%)	4 (11.4%)	29.18	<0.001**
>5 cm	34 (61.8%)	8 (30.8%)	26(89.7%)			3 (15%)	31 (88.6%)		
Implants									
Absent	20 (36.4%)	16 (61.5%)	4 (13.8%)	13.5	<0.001**	15 (75%)	5 (14.3%)	20.27	<0.001**
Present	35 (63.6%)	10 (38.5%)	25(86.2%)			5 (25%)	30 (85.7%)		
LVI									
Absent	30 (54.5%)	25 (96.2%)	5 (17.2%)	34.43	<0.001**	18 (90%)	12 (34.3%)	15.93	<0.001**
Present	25 (45.5%)	1 (3.8%)	24(82.8%)			2 (10%)	23 (65.7%)		
LN metastasis									
Negative	17 (30.9%)	15 (57.7%)	2 (6.9%)	16.56	<0.001**	15 (75%)	2 (5.7%)	28.61	<0.001**
Positive	38 (69.1%)	11 (42.3%)	27(93.1%)			5 (25%)	33 (94.3%)		
Distant metastasis									
Absent	43 (78.2%)	25 (96.2%)	18(62.1%)	9.337	0.002*	19 (95%)	24 (68.6%)	5.211	0.02*
Present	12 (21.8%)	1 (3.8%)	11(37.9%)			1 (5%)	11 (31.4%)		
Residual disease									
Absent	23 (41.8%)	17 (65.4%)	6 (20.7%)	11.25	0.001*	16 (80%)	7 (20%)	18.83	<0.001**
Present	32 (58.2%)	9 (34.6%)	23(79.3%)			4 (20%)	28 (80%)		
FIGO stage									
Stage IA	1 (1.8%)	1 (3.8%)	0 (0%)			1 (5%)	0 (0%)		
Stage IB	5 (9.1%)	5 (19.2%)	0 (0%)			5 (25%)	0 (0%)		
Stage IC	2 (3.6%)	2 (7.7%)	0 (0%)			2 (10%)	0 (0%)		
Stage IIA	4 (7.3%)	3 (11.5%)	1 (3.4%)	23.04	<0.001**	2 (10%)	2 (5.7%)	32.41	<0.001**
Stage IIB	5 (9.1%)	4 (15.4%)	1(3.4%)			5 (25%)	0 (0%)		
Stage IIIA	11 (20%)	6 (23.1%)	5 (17.2%)			2 (10%)	9 (25.7%)		
Stage IIIB	10 (18.2%)	2 (7.7%)	8 (27.6%)			1 (5%)	9 (25.7%)		
Stage IIIC	5 (9.1%)	2 (7.7%)	3 (10.3%)			1 (5%)	4 (11.4%)		
Stage IVA	9 (16.4%)	1 (3.8%)	8 (27.6%)			1 (5%)	8 (22.9%)		
Stage IVB	3 (5.5%)	0 (0%)	3 (10.3%)			0 (0%)	3 (8.6%)		

**, a highly significant difference (P<0.001); *, a significant difference (P<0.05).

**Table 3:** Relationship between ZIP-4 and ALDH1A1 Expression and Treatment Characteristics of the Studied Patients

	All	ZIP-4 expression				ALDH1A1 expression			
Variable		Low	High	Test^	p-value	Low	High	Test^	p-value
	(n=55)	(n=26)	(n=29)			(n=20)	(n=35)		
Chemotherapy									
No	5 (9.1%)	5 (19.2%)	0 (0%)	6.135	0.01*	5 (25%)	0 (0%)	9.625	0.002*
Yes	50(90.9%)	21(80.8%)	29 (100%)			15 (75%)	35(100%)		
Response to treatment	(n=32)	(n=9)	(n=23)			(n=4)	(n=28)		
Complete	3 (9.4%)	3 (33.3%)	0 (0%)			3 (75%)	0 (0%)		
Partial	2 (6.3%)	2 (22.3%)	0 (0%)	18.82	<0.001**	0 (0%)	2 (7.1%)	23.31	<0.001**
Stable disease	7 (21.8)	3 (33.3%)	4 (17.4%)			0 (0%)	7 (25%)		
Progressive disease	20 (62.5%)	1 (11.1%)	19 (82.6%)			1 (25%)	19 (67.9%)		
Relapse	(n=25)	(n=19)	(n=6)			(n=18)	(n=7)		
Absent	15 (27.3%)	14 (73.7%)	1 (16.7%)	6.17	0.01*	15(83.3%)	0 (0%)	14.58	<0.001**
Present	10 (18.2%)	5 (26.3%)	5 (83.5%)			3 (16.7%)	7 (100%)		
Type of relapse	(n=10)	(n=5)	(n=5)			(n=3)	(n=7)		
Platinum sensitive	4 (7.3%)	3 (60%)	1 (20%)	1.667	0.197	3 (100%)	1 (14.3%)	6.429	0.01*
Platinum resistant	6 (10.9%)	2 (40%)	4 (80%)			0 (0%)	6 (85.7%)		
Progression		(n=9)	(n=27)			(n=2)	(n=34)		
Absent	19 (34.5%)	0 (0%)	0 (0%)	0	1	0 (0%)	0 (0%)	0	1
Present	36 (65.5%)	9 (100%)	27 (100%)			2 (100%)	34 (100%)		
Mortality									
Alive	23 (41.8%)	21(80.8%)	2 (6.9%)	30.74	<0.001**	17 (85%)	6 (17.1%)	24.08	<0.001**
Died	32 (58.2%)	5 (19.2%)	27 (93.1%)			3 (15%)	29 (82.9%)		

^, Chi-square test; **, a highly significant difference (P<0.001); *, a significant difference (P<0.05)

**Table 4:** Relationship between ZIP-4 and ALDH1A1 Expression and Free Survival Time of the Studied Group

Variable	Low expression	High expression	Test	p-value
	ZIP-4	ZIP-4		
Disease-free survival (months)	(N=19)	(N=6)		
Mean ± SD	33.1 ± 4.04	20.6 ± 12.2	3.918	0.001*
Progression-free survival (months)	(N=9)	(N=27)		
Mean ± SD	12.8 ± 10.4	9.4 ± 6.8	1.152	0.257
Overall survival	(N=26)	(N=29)		
Mean ± SD	33.5 ± 6.4	22.9 ± 9.1	4.908	<0.001**
Variable	Low expression	High expression	Test	p-value
	*ALDH1A1*	*ALDH1A1*		
Disease-free survival (months)	(N=18)	(N=7)		
Mean ± SD	33 ± 7.1	22.5 ± 7.5	3.242	0.004*
Progression-free survival (months)	(N=2)	(N=34)		
Mean ± SD	4 ± 0	10.6 ± 7.9	-1.164	0.252
Overall survival	(N=20)	(N=35)		
Mean ± SD	33 ± 7.4	25.1 ± 9.5	3.209	0.002*

**, a highly significant difference (P<0.001); *, a significant difference (P<0.05)

**Table 5.** Relationship between ZIP-4 and ALDH1A1 Expression and Free Survival Time of the Studied Group

Variable	Median	95% CI	Test#	P-value
Disease-free survival ZIP-4				
Total	30.6 months	(27.2 – 34.1)	9.969	0.002*
Low expression	33.7 months	(31.8 – 35.7)		
High expression	20.6 months	(11.7 – 29.6)		
Disease-free survival ALDHA1				
Total	30.6 months	(27.2 – 34.1)		
Low expression	33.8 months	(30.5 – 37.1)	22.23	<0.001**
High expression	22.5 months	(17 – 28.1)		
Overall survival ZIP-4				
Total	27.9 months	(25.4 – 30.4)	31.99	<0.001**
Low expression	33.5 months	(31.1 – 35.9)		
High expression	22.9 months	(19.6 – 26.2)		
Overall survival ALDHA1				
Total	27.9 months	(25.4 – 30.4)	18.94	<0.001**
Low expression	33 months	(29.8 – 36.1)		
High expression	25.1 months	(21.9 – 28.1)		

#, Log-rank test; **, a highly significant difference (P<0.001); *, a significant difference (P<0.05)

## Author Contribution Statement

ASA, RS contributed to the study conception and design. All authors were responsible for the methodology and statistical analysis of data. ASA, RS, ABW, SH wrote the manuscript draft. All authors read and approved the final manuscript. 
